# Inflammatory Cytokines as Uremic Toxins: “Ni Son Todos Los Que Estan, Ni Estan Todos Los Que Son”

**DOI:** 10.3390/toxins9040114

**Published:** 2017-03-23

**Authors:** Esmeralda Castillo-Rodríguez, Soledad Pizarro-Sánchez, Ana B. Sanz, Adrian M. Ramos, Maria Dolores Sanchez-Niño, Catalina Martin-Cleary, Beatriz Fernandez-Fernandez, Alberto Ortiz

**Affiliations:** Department of Nephrology, IIS-Fundacion Jimenez Diaz, School of Medicine, Universidad Autonoma de Madrid; Fundacion Renal Iñigo Alvarez de Toledo-IRSIN and REDINREN, Av Reyes Católicos 2, 28040 Madrid, Spain; ecastillor@quironsalud.es (E.C.-R.); maria.pizarros@fjd.es (S.P.-S.); asanz@fjd.es (A.B.S.); amramos@fjd.es (A.M.R.); mdsanchez@fjd.es (M.D.S.-N.); cmartinc@fjd.es (C.M.-C.)

**Keywords:** chronic kidney disease, inflammation, uremic toxins, adipokines, chemokines, decoy receptor, mortality

## Abstract

Chronic kidney disease is among the fastest growing causes of death worldwide. An increased risk of all-cause and cardiovascular death is thought to depend on the accumulation of uremic toxins when glomerular filtration rate falls. In addition, the circulating levels of several markers of inflammation predict mortality in patients with chronic kidney disease. Indeed, a number of cytokines are listed in databases of uremic toxins and uremic retention solutes. They include inflammatory cytokines (IL-1β, IL-18, IL-6, TNFα), chemokines (IL-8), and adipokines (adiponectin, leptin and resistin), as well as anti-inflammatory cytokines (IL-10). We now critically review the cytokines that may be considered uremic toxins. We discuss the rationale to consider them uremic toxins (mechanisms underlying the increased serum levels and evidence supporting their contribution to CKD manifestations), identify gaps in knowledge, discuss potential therapeutic implications to be tested in clinical trials in order to make this knowledge useful for the practicing physician, and identify additional cytokines, cytokine receptors and chemokines that may fulfill the criteria to be considered uremic toxins, such as sIL-6R, sTNFR1, sTNFR2, IL-2, CXCL12, CX3CL1 and others. In addition, we suggest that IL-10, leptin, adiponectin and resistin should not be considered uremic toxins toxins based on insufficient or contradictory evidence of an association with adverse outcomes in humans or preclinical data not consistent with a causal association.

## 1. Introduction

“Ni son todos los que están, ni están todos los que son” is an old Spanish wordplay originally applied to insane persons and psychiatric hospitals in a theater play by poet Ramón de Campoamor (1817–1901). The wordplay revolves around the two different meanings of the verb “to be” in Spanish and thus, translation is difficult, but it could be roughly translated into “Not everyone who is in, should be in; neither everyone who should be in, is in”. This also applies to the current list of cytokines considered as uremic toxins or uremic retention solutes.

## 2. Inflammation in Chronic Kidney Disease

Chronic kidney disease (CKD) is among the fastest growing causes of death worldwide [1]. When the glomerular filtration rate (GFR) falls below 60 mL/min/1.73 m^2^, the risk of all-cause and cardiovascular death increases with decreasing GFR, peaking in patients undergoing dialysis [2]. This is thought to result mainly from accumulation of uremic toxins. Recent attention has focused on toxins that are not readily removed by dialysis procedures, such as protein-bound, gut-derived molecules [3]. In addition, markers of inflammation, like cytokines and adipokines, are associated with the risk of death in CKD patients, and are not efficiently removed by dialysis [4,5]. We now critically review the inflammation/uremic toxin interface and, specifically, cytokines that are considered uremic toxins or uremic retention solutes, with a focus on defining the potential clinical practice consequences.

## 3. Cytokines and Uremic Toxins

Cytokines are a broad and loose category of ~5–20 kDa extracellular cell signaling proteins that activate cell surface receptors, are mainly secreted by leukocytes and many mediate the inflammatory response [6]. Initially thought to be secreted only by leucocytes, it soon became obvious that most cells, including kidney parenchymal cells, secrete cytokines especially in response to stress. A molecule is considered a uremic toxin when two criteria are met, one related to the mechanism underlying its accumulation in CKD and another related to its contribution to CKD manifestations. Thus, uremic toxins have been defined as solutes normally excreted by the kidneys that are retained in CKD and interact negatively with biologic functions [7]. If no adverse consequences are known, they are termed uremic retention solutes. According to this narrow definition, cytokines are not strictly uremic toxins, since generally they are not excreted by the kidneys. However, as small proteins, they are filtered by normal glomeruli and uptaken and degraded by proximal tubules. Thus, cytokines may theoretically accumulate in kidney failure due to decreased degradation and may be encompassed by a wider definition of uremic toxins that involves defective molecule clearance in CKD rather than the narrower concept of decreased kidney excretion. Furthermore, in observational studies, higher circulating levels of diverse cytokines were associated with adverse clinical outcomes. These results are in accordance with cell culture and preclinical evidence of a deleterious effect of certain cytokines in tissue injury, including kidney or vascular disease. In this regard, a number of cytokines are cited among the 130 uremic toxins and uremic retention solutes in the European Uremic Toxin (EUTOX) Working Group database, the most comprehensive source on the subject [8,9]. Three categories of uremic toxins based on size and binding properties are recognized: free water-soluble low molecular mass (<0.5 kD) compounds, middle molecules (0.5–60 kD) and protein-bound solutes. According to the most recent EUTOX classification, a majority of identified uremic toxins and uremic retention solutes belong to the first category (68 molecules, 52%) and the rest are distributed between middle molecules (32 molecules, 35%), that includes cytokines, and protein-bound (30 molecules, 23%) compounds [9]. In the present review, we discuss the classification of cytokines as uremic toxins, and address the causes of cytokine accumulation in CKD, the evidence regarding their contribution to the consequences of renal failure and the therapeutic implications of these findings.

## 4. Cytokines as Uremic Toxins

Cytokines already represented 4 of 90 (4.4%: IL-1β, IL-6, TNFα and the adipokine leptin) uremic retention solutes in the first published EUTOX uremic toxin database in 2003 [7]. The number increased to 9 of 130 (7%) uremic toxins and uremic retention solutes in the most recent 2012 database [9] (Table 1). Four are inflammatory cytokines (IL-1β, IL-18, IL-6, TNFα), one is a chemokine (IL-8), IL-10 is considered an anti-inflammatory cytokine, and adiponectin, resistin and leptin are adipokines, that is, cytokines secreted mainly, but not exclusively, by adipocytes [9,10]. IL-8 is one of 21 uremic toxins with serum concentration in uremia >10-fold higher than normal values [9].

### 4.1. IL-1β and IL-18

Both IL-1β and IL-18 have a complex regulation. Secretion of the active cytokine requires increased mRNA expression and intracellular pro-cytokine levels as well as inflammasome-mediated activation of caspases that cleave the precursors to yield the active cytokine that is then secreted. As a result, despite mRNA expression in many cell types, only macrophages release significant amounts of IL-1β, often within the cell death process called pyroptosis [11]. In contrast, IL-18 is also released by enterocytes and stressed tubular cells, among others. IL-1β was the first interleukin described and is an extremely pro-inflammatory molecule, which circulates at very low concentrations in healthy individuals. In humans, excessive IL-1β secretion is known to cause disease and several therapeutic strategies are available to target IL-1β in inflammatory conditions such as rheumatoid arthritis, including the IL-1β receptor antagonist (IL-1ra) anakinra [4]. IL-1ra belongs to the IL-1β superfamily but binds to the IL-1β receptor non-productively in competition with IL-1β. Thus, the biological activity of IL-1β depends both on the levels of IL-1ra and of IL-1β. In this regard, circulating endogenous IL-1ra levels are also increased in CKD [12]. Since both the active cytokine and its antagonist are increased in CKD, proof is needed that the concentrations of IL-1β found in CKD are indeed deleterious. In a proof-of-concept study, anakinra given for 4 weeks to inflamed hemodialysis patients decreased hsCRP by 53% and IL-6 by 40% compared with a 20% increase in the placebo arm [13]. Thus, there is evidence that IL-1β contributes to inflammation in CKD. Whether anakinra administration improves survival or improves other clinical outcomes in CKD patients is currently unknown and additional long-term studies should be performed to settle the issue.

IL-18 is listed as a uremic retention solute in the EUTOX database [8] but not in the recent EUTOX publication, which lists IL-8 but, surprisingly, not IL-18 [9]. IL-18 promotes tissue injury such as kidney fibrosis and vascular inflammation in preclinical studies [14,15]. In this regard, serum IL-18 predicted 2-year cardiovascular mortality in patients at various stages of CKD with a past history of acute myocardial infarction [16]. Iboctadekin (recombinant IL-18) was studied in early clinical trials to treat malignancy but development was discontinued for lack of efficacy, although tolerance was good [17]. Discontinuation of clinical development means that no further information of its effect in humans will become available.

### 4.2. IL-6

IL-6 is a proinflammatory cytokine mainly secreted by macrophages. IL-6 binds to the IL-6 receptor (IL-6R), which interacts with the signal-transducing gp130 receptor, leading to Janus kinase/Signal Transducers and Activators of Transcription (JAK/STAT) signaling [18]. Contrary to other soluble receptors, which function as decoy receptors, soluble IL-6R (sIL-6R) facilitates IL-6 signaling. Conventional signaling involves binding of IL-6 to transmembrane IL-6R on cells expressing this receptor, while trans-signaling involves binding of the sL-6R/IL-6 complex to membrane-bound gp130. Trans-signaling allows IL-6 to elicit biological effects in cells lacking IL-6R. This may explain apparently opposite actions in tissue injury, e.g., pro-inflammatory and regenerative in acute kidney injury [19]. In contrast, soluble gp130 (sgp130) is a decoy receptor. Thus, concentrations of sIL-6R and sgp130 are as important as concentrations of IL-6 to determine actual IL-6 biological activity. In dialysis patients, sIL-6R levels are increased [20], while sgp130 are higher in hemodialysis patients using poorly biocompatible membranes than in controls, but are not elevated in other ESRD patients [21]. As a consequence, higher IL-6 conventional and trans-signaling is expected in CKD. Indeed, both IL-6 and sIL-6R are independent predictors of mortality in patients with ESRD and among cytokines, IL-6 has the highest predictive value for patient death [20,22]. Thus, sIL-6R should also be studied as a potential uremic toxin.

IL-6 has a number of actions that are potentially deleterious in CKD patients. It is a key driver of the inflammatory response, the liver acute reactant response and cancer-induced cachexia [23]. Infusion of IL-6 in humans increased resting energy expenditure, plasma free fatty acids and fat oxidation [24]. Because of its involvement in disease, several agents targeting IL-6 are in clinical use or in the preclinical pipeline [4,25]. Siltuximab is an anti-IL-6 and tocilizumab is anti-IL-6R monoclonal antibody. Both interfere with both classical and trans IL-6 signaling. Indications include multicentric Castleman disease and rheumatoid arthritis. Despite the availability of these agents, no trials have addressed whether targeting IL-6 improves outcomes in CKD or in patients with CKD treated for other reasons, thus demonstrating its role as a uremic toxin. A Mendelian randomization approach was consistent with a role of IL6R in coronary heart disease [26] and clinical trials are underway testing anti-IL-6 agents for cardiovascular diseases, but not in the CKD context [27].

### 4.3. TNFα

Tumor Necrosis Factor-alpha (TNFα) is a key pro-inflammatory cytokine synthesized in large amounts by activated macrophages and T cells, but also by stressed epithelial and other cells [28]. TNFα stimulates the release of inflammatory cytokines (IL-1β, IL-6, IL-8, and others), upregulates the expression of endothelial adhesion molecules and chemokines (MCP-1, CXCL16 and others), promotes cell death and decreases the expression of the anti-inflammatory and anti-aging protein Klotho [29,30]. Infusion of TNFα decreases free fatty acids and increases glucose appearance without modifying energy expenditure [31]. TNFα also promotes vascular calcification, although its role in uremia-associated vascular calcification has not been addressed in vivo [32,33,34].

In CKD patients, high levels of TNFα are associated with markers of malnutrition and inflammation and predict mortality, although it is a weaker predictor of mortality than IL-6 [22,35,36,37].

TNF is pathogenic in a number of preclinical conditions including kidney disease [38,39]. Anti-TNFα therapy is currently in clinical use for inflammatory conditions, such as rheumatoid arthritis, the seronegative spondyloarthropathies, and inflammatory bowel disease. Agents include anti-TNFα monoclonal antibodies (infliximab, adalimumab, golimumab), antigen-binding fragment (Fab’: certolizumab) and a soluble TNFα receptor (sTNFR: etanercept) [40]. Interestingly, in patients with rheumatoid arthritis and CKD the use of anti-TNF therapies was associated with slower loss of GFR, although it is unclear whether this is the result of interfering with rheumatoid arthritis disease activity or with CKD-associated progression of kidney injury [41]. In CKD, circulating levels of TNFα are well within the range found in rheumatoid arthritis patients treated with infliximab [42]. A recent trial aimed at studying whether the TNF receptor antagonist etanercept was safe and improved albumin and prealbumin levels in inflamed hypoalbuminemic (albumin <3.8 g/dL, CRP >8.0 mg/L) prevalent hemodialysis patients. However, recruitment was marred by the low prevalence of this combination, less than 6% of 433 screened patients met the inclusion criteria. In the 10 randomized patients, etanercept for 44 weeks was safe and had significant time-dependent effects on prealbumin [43].

Development of etanercept mimicked a physiological phenomenon: the occurrence of soluble cytokine receptors that behave as decoy receptors for inflammatory cytokines by competing for cytokine binding with cell membrane receptors [44]. sTNFR1 and sTNFR2 are the circulating forms of the membrane–bound, signal transducing TNFR1 and TNFR2 receptors and, like etanercept, they inhibit TNFα activity. Interestingly, serum levels of both sTNFR are elevated in CKD patients and inversely correlate with GFR [45]. Both sTNFR1 and sTNFR2 predict CKD progression, risk of death and cardiovascular events in advanced CKD patients [45,46,47,48,49]. Since sTNFRs are thought to protect from TNFα-mediated tissue injury, this observation may be interpreted as a note of caution towards moving from an observational association to causality, as it has been done for other cytokines. However, the issue is more complex. sTNFR1 and sTNFR2 are well in excess, 90- and 130-fold respectively, of TNFα in the circulation of CKD patients [47]. In fact, free TNFα (3–10 ng/L) is only around 30% of total circulating TNFα and three orders of magnitude below the concentrations of TNFα used in cell culture experiments to assess TNFα biological activity. In mice, bilateral nephrectomy results in an increase of the levels of both sTNFR and TNFα, but the increased TNFα is biologically inactive as it is for the most part bound to sTNFR [50]. This raises doubts about the pathogenic potential of increased circulating TNFα in the course of CKD and its consideration as a uremic toxin. On the other hand, anti-TNF therapies have been marred by an increased incidence of infections, especially tuberculosis [51]. Since CKD is also associated with impaired defense against mycobacterium tuberculosis [52], consideration should be given to add sTNFR to the list of uremic toxins. In addition, the use of anti-TNF strategies is associated with increased risk of venous thromboembolic events [53], also more frequent in CKD patients [2], and clinical development was discontinued for several indications because of increased mortality or safety concerns [54].

### 4.4. IL-8

Interleukin-8 (IL-8, CXCL8) was the first chemoattractant cytokine (chemokine) discovered, in 1978. It is released by macrophages, endothelial and epithelial cells, and recruits neutrophils to sites of infection or tissue injury. IL-8 and/or its receptors CXCR1 and CXCR2 also have tumorigenic and proangiogenic properties [55,56]. Thus, high IL-8 levels may potentially contribute to inflammatory and tumor complications of CKD [2].

IL-8 is considered a uremic retention solute in the recent EUTOX publication [9], but, surprisingly, not in the EUTOX database [8]. In hemodialysis patients, higher IL-8 levels correlated with increased all-cause and cardiovascular mortality [57].

Clinical development of anti-IL8 for psoriasis and pulmonary disease was discontinued. However, clinical development continues for malignancy and for palmoplantar pustulosis induced by EGFR inhibitors with positive results and good tolerability, and may help further understand the biology of this cytokine in humans [58,59].

### 4.5. IL-10

IL-10 is a pluripotent cytokine produced by many activated immune cell types, including T-helper (Th2) cells, B cells, macrophages, monocytes, and by epithelial cells. It is a key factor to limit and shut down the inflammatory response and to switch from tumor-promoting inflammation to antitumor immunity [57,58,59,60]. IL-10 suppresses the production of proinflammatory mediators and downregulates co-stimulatory molecules required for T cell activation. It also inhibits activation of macrophages and other cells [61]. IL-10 deficiency leads to stimulation of inflammatory responses, inflammatory bowel disease and spontaneous tumor development [60]. In this regard, IL-10 stimulates cytotoxicity of CD8+ T cells promoting tumor rejection [60]. In animal models, IL-10 had beneficial anti-inflammatory effects in the kidney and other organs [61]. Indeed, recombinant human IL-10 has been tested in clinical trials to treat inflammatory conditions such as vasculitis, intestinal inflammatory disease, and Behçet disease as well as for advanced cancer [62]. IL-10 reduced disease-associated proinflammatory cytokines, but disease activity was not significantly modified [61]. Treatment-related adverse events included anemia and thrombocytopenia [62,63]. These may be present in CKD, but it is unclear whether only pharmacological IL-10 doses elicit these effects, as they were dose-dependent [63]. There are no studies in the CKD or ESRD population.

Higher serum IL-10 levels in CKD patients have been associated with the risk of cardiovascular events and mortality during follow-up [64,65]. This is paradoxical with the known functions of IL-10. The elevation of IL-10 in CKD has been considered an adaptive, counter-regulatory mechanism to control uremia- and dialysis-induced inflammation, that nevertheless results insufficient to completely shut them off [66]. Peter Stenvinkel labeled it “the good” [35]. In this regard, stratification for the IL-10 −1082 polymorphism, which determines the amount of IL-10 produced following stimulation, revealed a higher risk for cardiovascular events and mortality for the “low-producing” genotype [66]. Thus, it is unclear that IL-10 should be considered a uremic toxin, as there is little evidence that the levels found in uremia are detrimental. Furthermore, it raises questions about the consideration of a molecule as a uremic toxin based on observational association studies in humans.

## 5. Adipokines

Adipokines are cytokines secreted preferentially by adipocytes. A recent review suggested that, based on functional animal data and observational human studies, only leptin may be considered as a full uremic toxin, while resistin and visfatin display some features of uremic toxins, and, in contrast, high levels of adiponectin and chemerin seen in uremia appear to be beneficial [67]. The EUTOX collaboration had a different criterion, however, identifying adiponectin, leptin and resistin, but not visfatin and chemerin, as uremic retention solutes [9]. The lack of functional human studies in the CKD context and contradictory observational studies underlie these differences in the evaluation of the available evidence.

### 5.1. Adiponectin

Adiponectin is cleared by the kidneys, but the inflammatory environment associated to uremia impairs adiponectin synthesis [68]. Patients with renal disease have 2–3 fold higher plasma adiponectin levels, without changes in adiponectin fractions or in adiponectin receptors [67].

Adiponectin has anti-atherogenic, anti-inflammatory, nephroprotective and cardioprotective actions in preclinical models. Indeed, low plasma adiponectin levels have been associated with obesity, hypertension, type 2 DM and coronary disease [67,69]. However, in the CKD context, there are no interventional studies in humans administering adiponectin or inhibiting adiponectin. Thus, it is currently unclear whether higher adiponectin levels in CKD are contributing to a more severe or less severe CKD phenotype. Indeed, information from observational studies is controversial. Higher adiponectin levels have been associated with both worse and better outcomes. In observational studies in CKD patients, high circulating adiponectin levels are associated with higher mortality [70,71] while lower or higher adiponectin levels associated to increased mortality and cardiovascular events in dialysis patients [72,73,74,75].

### 5.2. Leptin

Leptin is secreted by white adipose tissue and regulates energy balance by decreasing food intake (the “satiety” hormone) and enhancing energy expenditure [76]. Obese patients have markedly elevated serum leptin levels. Leptin levels are higher in CKD patients than in non-CKD patients matched for body mass index, but still correlate with body fat in CKD [77].

Leptin has pro-fibrotic effects, promoting TGFβ1 synthesis, glomerulosclerosis and renal damage [78], atherosclerosis and VSMC hypertrophy [79], angiogenesis [80] and platelet dysfunction [81]. Leptin increased blood pressure in animals [82] and stimulated secretion of the phosphaturic hormone FGF-23 [83], while a pegylated leptin receptor antagonist or deficiency of the hypothalamic melanocortin receptor 4 (MC4-R) decreased anorexia in uremic mice [84,85]. It has been speculated that leptin may contribute to protein-energy wasting, cardiovascular disease and inflammation in human CKD [67]. However, no functional studies in humans have addressed these hypotheses. Clinical studies have not demonstrated conclusive results about the potential role of leptin in cachexia and lean body mass loss, and low leptin levels were associated with mortality in dialysis patients but not in all studies [86,87,88,89,90,91]. Leptin and its analog metreleptin are in clinical use to treat congenital leptin deficiency and generalized lipodystrophy.

The somewhat contradictory information on the relationship between adiponectin, leptin and mortality may depend on the interaction of the risk provided by these adipokines and abdominal obesity. A fixed excess of leptin was associated with increasing risk for all-cause and cardiovascular mortality in patients with abdominal obesity but had the opposite effect in those without abdominal obesity. Adiponectin did not predict mortality when leptin was considered as confounder [92].

### 5.3. Resistin

Resistin is secreted by adipose tissue and promotes insulin resistance in mice while in humans, monocyte/macrophages are the key sources and it promotes inflammation [93]. Thus, although frequently listed as an adipokine, there is little evidence that it is an adipokine in humans. Resistin is now considered a biomarker of inflammation but not of insulin resistance in CKD [94,95]. High circulating resistin levels are associated with mortality risk among patients with diabetes and coronary artery disease [96]. However, no clear-cut association to mortality was observed in CKD patients, in whom the relationship to mortality depended on adiponectin levels [97]. Thus, there is no firm evidence to classify resistin as a uremic toxin.

## 6. Which Additional Cytokines Should Be in Listed as Potential Uremic Toxins?

Circulating levels of a host of other cytokines and chemokines are increased in CKD patients, including, but not limited to IL-2, IL-4, IL-5, IL-12, CCL2, CCL5, CXCL12, CX3CL1 and CXCL16 [36,98,99]. Some of them have been associated to CKD-related symptoms or adverse outcomes. IL-2, a cytokine that induces pruritus when administered to humans, was associated to pruritus in dialysis patients [100,101]. CXCL12 was associated with incident myocardial infarction and death in CKD patients [102]. In the same patient cohort, CX3CL1 plasma levels were associated with risk of cardiovascular disease and death [103]. Both CXCL12 and CX3CL1 promote atherosclerosis in preclinical models. CCL2 and CCL5 contribute to kidney inflammation and fibrosis [104]. Indeed, they are being explored as clinical targets in diabetic kidney disease. CCX140-B, an inhibitor of the CCR2 receptor for CCL2, and the CCL2 inhibitor emapticap pegol were among the most promising drugs for diabetic kidney disease in phase II RCTs [105]. In addition, there is some suggestion that circulating CCL2 is associated with adverse outcomes in dialysis patients [106]. Circulating CXCL16 is also increased in CKD and there is preclinical evidence that it may cause kidney and vascular injury, although human studies linking this chemokine to adverse outcomes in CKD are lacking [107,108,109].

An interesting aspect is that not all circulating cytokines are increased in CKD. As an example, circulating TNF-related weak inducer of apoptosis (TWEAK), levels are decreased in CKD [110,111,112]. This is surprising given the small size of TWEAK. TWEAK is a pleiotropic cytokine that promotes kidney and vascular injury through activation of the Fn14 receptor [113,114]. Interestingly, inflammation increases the expression of the Fn14 receptor, sensitizing cells to TWEAK action. In this regard, even though circulating TWEAK levels are lower in CKD, CKD patients that have higher TWEAK levels than other CKD patients are at increased risk of death, especially if there is evidence of systemic inflammation, that is, if Fn14 expression is expected to be high [111].

Chemerin and visfatin are additional adipokines, which circulate at higher concentration in CKD patients [68], although there is contradictory information regarding their association to adverse outcomes. Circulating chemerin levels were increased in CKD and dialysis patients, and they were associated with worse cardiovascular outcomes in CKD but with better outcomes in dialysis patients [115,116,117]. Lack of specific assays for active chemerin may contribute to the confusion, since there is preclinical evidence of a deleterious effect of chemerin on vascular cells [118]. Visfatin/Nicotinamide phosphoribosyltransferase (NAMPT) is both an adipokine and an intracellular protein. Serum visfatin was increased in CKD and was associated with progressive loss of GFR and mortality, although the association with mortality was dependent on its association with inflammatory markers [119,120]. Although frequently described as a proinflammatory adipokine, in diabetic nephropathy increased visfatin production plays an adaptive role, dampening inflammatory and lethal responses in tubular cells [121].

## 7. Conclusions and Future Directions

The data suggest that consideration should be given to remove some cytokines from lists of uremic toxins, while adding others (Table 2). Those removed may still be classified as uremic retention solutes. As an example, IL-10 has multiple potentially beneficial effects and in fact, has been tested as an anti-inflammatory agent in human inflammation. It is likely that its association with adverse outcomes results from IL-10 levels being high but not high enough to compensate the increase in other mediators of inflammation. The insufficient recruitment of compensatory adaptive mechanisms is a current topic of research that may mislead into attributing a pathogenic role to protective molecules when only observational studies are considered [122]. For other cytokines, the evidence linking the protein to CKD complications is controversial from an observational point of view and contradictory from a preclinical functional point of view. As an example, adiponectin has a number of beneficial actions that cast into doubt that high levels may contribute to CKD manifestations. In contrast, a growing list of cytokines and related molecules are found increased in CKD, there is preclinical evidence of potential contribution to disease in several organs and these cytokines have been associated in observational studies to adverse outcomes in CKD. Thus, they fulfill the requirements to be considered uremic toxins. The myriad of cell culture or animal studies may create confusion by testing non-relevant concentrations of cytokines or addressing therapeutic effects related to high local concentrations and not the potential adverse effects of the circulating cytokine. In this regard, only in vivo targeting in humans of specific cytokines may confirm or discard that a specific cytokine is a uremic toxin in the sense that it contributes to CKD manifestations. Cytokines are especially interesting in this regard for two reasons. First, there are biological agents that target cytokines in clinical development or already in clinical use. The use of these biologicals targeting cytokines provided proof-of-concept evidence for a role of TNFα and IL-1β in CKD manifestations. Second, cytokines are relatively large molecules and cytokine clearance by renal replacement therapy techniques remains low, in some cases barely removing the excess cytokine secreted during the dialysis procedure. In a recent study, IL-6 clearance was around 25 mL/min in a 4 h hemodialysis session, that is, 1 mL/min over a 72 h period of which dialysis is performed for just 4 h [123]. This was 3 times lower than β_2_-microglobulin, a protein less than half its size, and was not enough to compensate the increased production of IL-6 associated with the dialysis procedure. Thus, IL-6 concentration was unchanged or slightly increased following dialysis, depending on the membrane used [123]. In previous studies, clearance of IL-6 was lower than clearance of IL-1β or IL-1RA, similar to clearance of IL-8 and roughly double the clearance of TNFα [124,125].

**Figure 1:**
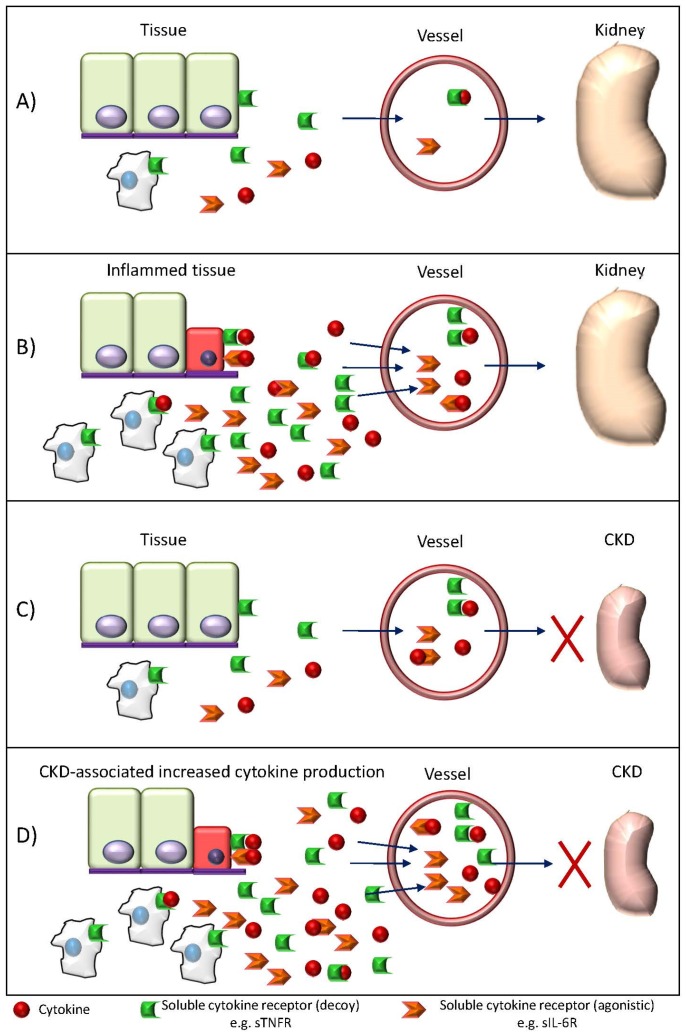
Increased production versus decreased clearance of cytokines that are potential uremic toxins in CKD: practical consequences. Traditionally, increased levels of uremic toxins, such as cytokines, were considered to result from decrease renal clearance. However, there is evidence for increased production of certain uremic toxins, such as PTH or cytokines. The distinction may have therapeutic implications. (**A**) Healthy subject. A small amount of cytokines and soluble cytokine receptors are produced and cleared by the kidneys and other organs. Soluble decoy receptors will render cytokines inactive, while agonistic soluble receptors will allow transactivation of target cells not expressing the receptor in the cell membrane; (**B**) Inflammatory disease with normal renal function. Increased local production of cytokines and receptors in the inflamed tissue will lead to leukocyte infiltration and tissue injury (the red epithelial cell represents a damaged cell), as well as to increased circulating levels of cytokines and soluble receptors. Sterile inflammatory conditions are currently treated with anti-cytokine strategies; (**C**) Conventional view of uremic toxins, including cytokines, in CKD. Decreased kidney clearance will lead to higher circulating levels. This may be deleterious for the vasculature, but tissue levels are not expected to reach the values found in inflammatory conditions. However, the ultimate consequences of the high cytokine levels will depend on the levels of modifiers such as decoy and transactivating soluble receptors; (**D**) Current view of cytokines as uremic toxins. On top of the reduced renal clearance, there is an excessive production of cytokines and soluble receptors, leading to higher tissue cytokine concentrations and tissue injury. Both leukocytes and parenchymal cells secrete excess amounts of cytokines in response to the presence of other uremic toxins. The excess production of cytokines will render efforts at increasing cytokine clearance useless, as suggested by unchanged cytokine levels during dialysis despite clearance. These patients may benefit from exploring anti-cytokine strategies. In individual patients, the scenario presented in (**C**) or (**D**) may predominate, depending among other factors, on levels of uremic toxins that are known to promote cytokine secretion and are modulated by environmental factors or by diet and the gut microbiota, such as p-Cresyl sulphate.

A second issue that merits discussion is to what extent cytokines fulfill the requirements to be considered uremic toxins from the point of view of the mechanisms leading to cytokine accumulation in CKD. The fate of cytokines in vivo, especially in the physiological range of concentrations, is poorly understood. Thus, the concept that the increased levels of cytokines in CKD patients are primarily the result of lower kidney clearance may be questioned. Pharmacokinetics studies may be biased by the use of high dose of cytokines. Pharmacokinetics studies in rats and mice have identified the kidney and liver as major clearance organs for IL-1β, IL-6 and TNF-α, although other organs also contribute and the half-life is in the order of minutes, while the kidney is a major site for leptin clearance [126,127,128,129,130,131]. For IL-6, up to 80% may be cleared by the rat liver [132]. In humans on dialysis, the pharmacokinetics of low dose IL-2 and changes in IL-6 and TNF-α were essentially similar in all patients studied, including one anephric patient, irrespective of the periods of dialysis [133]. However, higher doses of IL-2 may have renal clearance and this was attributed to saturation of physiological clearance mechanisms. In contrast, there is evidence supporting a role of increased cytokine production on cytokine accumulation in uremia, which may be secondary to the accumulation of other uremic toxins. Thus, as uremic toxins promote cytokine expression and release, there is a relationship between serum levels of certain toxins and cytokine levels in CKD, and hemodialysis clearance of cytokines is not associated to a reduction in serum cytokine levels [123,134,135]. As an example, serum indoxyl sulfate was independently associated with serum IL-6, TNF-α and IFN-γ, while p-cresyl sulphate was independently associated with serum IL-6 and these toxins increase the gene expression of cytokines and chemokines [134,135]. In inflamed hemodialysis patients, anakinra decreased IL-6 by 40%, suggesting that increased IL-6 synthesis may contribute around 40% of circulating IL-6 this population [13,136]. A uremic environment enhances TNFα release from peripheral blood leukocytes [137]. Exposure of endothelial cells to uremic plasma results in a time- and CKD-stage-dependent increased expression of chemokines IL-8 and MCP-1 [138]. Leptin production is increased in adipocytes in uremic conditions [139,140] and in mice, uremia increases resistin expression [141]. Leptin is also overproduced by white adipose tissue in uremic conditions and oversecretion could contribute to hyperleptinemia observed in CKD [139,140]. This is not merely an academic discussion (Figure 1). If indeed increased cytokine production is a key driver of increased circulating cytokine levels in uremia, we may expect higher local concentrations at the sites of cytokine secretion than plasma concentrations. In these circumstances, an increased clearance of cytokines will not decrease local cytokine concentrations and the focus should be in decreasing their synthesis or inhibiting their activity. Understanding the regulators of cytokine secretion may identify key transcription factors such as NF-κB, which may be targeted to decrease the concentrations of several cytokines [142,143].

In conclusion, it is likely that the current list of cytokines that are considered potential uremic toxins is both incomplete (ni están todos los que son) and excessive (ni son todos los que están). Indeed, new molecules should be also considered as potential uremic toxins, such as ZAG (Zinc alpha-2-glycoprotein), which is increased in CKD and contributes to cancer cachexia [144,145]. The potential of cytokines to be targeted by biologicals makes understanding their role in the uremic syndrome a key priority. Additionally, an improved understanding of the factors leading to cytokine accumulation is CKD may help focus our attention in trying to increase their clearance or decrease their synthesis.

## Figures and Tables

**Table 1:** Cytokines currently considered potential uremic toxins. β2-microglobulin is used as a well characterized comparator from the point of view of renal and dialyzer clearance.

	Normal Concentration * (ng/L)	Uremic Concentration (ng/L) *	Relative Increase	MW (kD)	Targeting at Clinical Development Stage
**Inflammatory cytokines and chemokines**					
**Interleukin-1β**	160	236 ± 92	1.5	32	In use
**Interleukin-18**	142 ± 48	202 ± 169	1.4	20	Yes
**Interleukin-6**	4.0	5.9 ± 2.0	1.5	24.5	In use
**Tumor Necrosis Factor α (TNFα)**	7.0	21.6 ± 24.5	3.0	26	In use
**Interleukin-8**	1.64 ± 1.85	20.2 ± 25.1	10.9	8	Yes
**Anti-inflammatory cytokines**					
**Interleukin-10**	7.10 ± 1.50	10.60 ± 6.00	1.5	18	Yes
**Adipokines**					
**Adiponectin**	8,700,000 ± 4,800,000	16,600,000 ± 6,600,000	2.0	28	No
**Leptin**	8400 ± 6700	37,600 ± 25,100	4.5	16	In use
**Resistin**	15,100 ± 700	47,300 (35,300–62,200)	3.1	12.5	No
**Comparator**					
**β2-microglobulin**	1,900,000 ± 1,600,000	30,200,000 ± 7,800,000	15.9	11.8	NA

* Average values expressed as mean ± SD or (range); MW: Molecular weight; NA: not applicable; Data from references [8,9]. When discrepant, reference [9] data were used.

**Targeting at Clinical Development Stage**: In use: medication in routine clinical use; Yes: medication in clinical development; No: medication not in clinical development.

**Table 2:** Cytokines as potential uremic toxins: evidence supporting the consideration of cytokines as uremic toxins.

Those Already in	Evidence for role in Human CKD-Associated Abnormalities	Comment	Consider Removing from List of Potential Uremic Toxins *	Comment	Consider Adding to List of Potential Uremic Toxins	Comment
**IL-1β**	Anakinra: IL-1β promotes inflammation	Increased decoy receptors may be protective	**IL-10**	Anti-inflammatory effect	**IL-6R**	Associated to mortality, facilitates IL-6 signaling
**IL-18**	Only observational		**Adiponectin**	Insufficient evidence in humans	**IL-2**	Causes pruritus, associated to pruritus
**IL-6**	Only observational	Increased soluble receptor may increase some effects	**Leptin**	Insufficient evidence in humans	**sTNFR1, sTNFR2**	Associated to mortality, may sensitize to tuberculosis in humans
**TNFα**	Anti-TNF: TNF may contribute to CKD progression and malnutrition	Increased decoy receptors may be protective	**Resistin**	Insufficient evidence in humans	**CXCL12**	Associated to mortality,
**IL-8**	Only observational				**CX3CL1**	Associated to mortality,
**IL-10**	Only observational					
**Adiponectin**	Inconclusive observational	Association with mortality inconsistent and related with either low or high levels				
**Leptin**	Inconclusive observational	Low leptin levels associated with mortality in some populations				
**Resistin**	Inconclusive observational	Association with mortality inconsistent				

* Given the high plasma values, they would still be considered uremic retention solutes.
